# Complete Genome Sequences of Porcine Epidemic Diarrhea Virus Strains JSLS-1/2015 and JS-2/2015 Isolated from China

**DOI:** 10.1128/genomeA.01200-16

**Published:** 2016-11-10

**Authors:** Jie Tao, Benqiang Li, Chunling Zhang, Huili Liu

**Affiliations:** a Institute of Animal Science and Veterinary Medicine, Shanghai Academy of Agricultural Sciences, Shanghai, China; b Municipal Key laboratory of Agri-Genetics and Breeding, Shanghai, China

## Abstract

Two porcine epidemic diarrhea virus (PEDV) strains, JSLS-1/2015 and JS-2/2015, were isolated from piglets with watery diarrhea in South China. Two genomic sequences were highly homologous to the attenuated DR13 strain. Furthermore, JSLS-1/2015 contains a 24-amino-acid deletion in open reading frame 1b, which was first reported in PEDV isolates.

## GENOME ANNOUNCEMENT

Porcine epidemic diarrhea (PED) is a highly contagious disease characterized by vomiting, watery stool, and dehydration in suckling pigs that is caused by porcine epidemic diarrhea virus (PEDV) (1, 2). PEDV was first reported in England in 1971, and sporadic cases were reported throughout Europe until the end of the 1990s (3, 4). In 2010, a large-scale outbreak of pathogenic-variant PEDV emerged in pig herds in China, with 80 to 100% morbidity and 50 to 90% mortality (5–7).

In October 2014, porcine fecal samples were taken from piglets with diarrhea at different swine-raising farms in South China where bi-combined killed or attenuated vaccines against transmissible gastroenteritis virus and PEDV infection were used. Positive samples confirmed by reverse transcription PCR using specific primers against the PEDV M gene were inoculated on Vero cells for succession cultivation. Viral RNA of the 40-s passaged viruses were extracted using Trizol (Invitrogen, USA) according to standard protocol. Twenty-five pairs of primers overlapping the whole PEDV genome were designed for sequencing the complete PEDV genomes (8). Sequences at the 5′-end of the isolates were obtained with the 5′ RACE kit, and those at the 3′-end were amplified using an Oligo(dT) primer with an adapter at the 5′-terminal. All of the sequencing reactions were done in duplicate, and all sequences were confirmed by sequencing both strands.

The full-length sequences of JSLS-1/2015 and JS-2/2015 contained 27,861 and 27,930 nucleotides (nt), respectively. The complete genomic sequence identity of the two PEDV isolates reached 99.9%, and their genomic organizations were found to be the same as the attenuated strain DR13(JQ0233162). However, there was a 24-amino-acid (aa) deletion in open reading frame 1b (ORF1b) of strain JSLS-1/2015. This differs from strain HUA-14PED96, which contained a 72-nt deletion in ORF1a. We cannot be sure whether the deletion in ORF1b is attributed to the virulence of the isolate. Besides, the isolates had the same aa deletions as strain CV777 at aa sites 59 to 62 and at sites 141 and 163 in the S gene and showed 94.8% to 99.7% homologies to the PEDV strains in the G1 group. Comparison with previously published M gene reports in China showed that JSLS-1/2015 and JS-2/2015 are most closely related to strains HLJ-2012 (99.8%) and BJ-2012-1 (99.9%), respectively. Moreover, N gene alignment reports indicated that the sequences of the two strains had identities above 99.5% with the attenuated DR13 strain, and E gene homology analysis showed a 100% identity between JSLS-1/2015 and the attenuated DR13 strain and 99.5% identity between JS-2 and the attenuated DR13 strain. Further analysis of the genetic evolution of the ORF3 genes found that there were 51 nt deleted in both the JSLS-1/2015 and JS-2/2016 strains, which is a common feature among the attenuated strains.

To our knowledge, this is the first report of a complete PEDV sequence isolated from dead pigs with a long-fragment deletion in ORF1b. So, further studies will be carried out to explore the virus’s biological activities and pathogenesis in causing disease comprehensively.

### Accession number(s).

The complete genome sequence of PEDV strains JSLS-1/2015 and JS-2/2015 have been submitted to GenBank under the accession numbers KX534205 and KX534206, respectively.
